# Safety and feasibility of laparoscopic radical resection for bismuth types III and IV hilar cholangiocarcinoma: a single-center experience from China

**DOI:** 10.3389/fonc.2023.1280513

**Published:** 2023-12-18

**Authors:** Jianjun Wang, Yang Xia, Yuan Cao, Xintao Zeng, Hua Luo, Xianfu Cai, Mingsong Shi, Huiwen Luo, Decai Wang

**Affiliations:** ^1^Department of Hepatobiliary Surgery, Mianyang Central Hospital, School of Medicine, University of Electronic Science and Technology of China, Mianyang, China; ^2^Department of Neurosurgery, Mianyang Central Hospital, School of Medicine, University of Electronic Science and Technology of China, Mianyang, China; ^3^Department of Urology, Mianyang Central Hospital, School of Medicine, University of Electronic Science and Technology of China, Mianyang, China; ^4^National Health Commission (NHC) Key Laboratory of Nuclear Technology Medical Transformation, Mianyang Central Hospital, School of Medicine, University of Electronic Science and Technology of China, Mianyang, China

**Keywords:** hilar cholangiocarcinoma, laparoscopy, radical resection, safety, caudate lobectomy

## Abstract

**Background:**

Surgery represents the only cure for hilar cholangiocarcinoma (HC). However, laparoscopic radical resection remains technically challenging owing to the complex anatomy and reconstruction required during surgery. Therefore, reports on laparoscopic surgery (LS) for HC, especially for types III and IV, are limited. This study aimed to evaluate the safety and feasibility of laparoscopic radical surgery for Bismuth types III and IV HC.

**Methods:**

The data of 16 patients who underwent LS and 9 who underwent open surgery (OS) for Bismuth types III and IV HC at Mianyang Central Hospital, School of Medicine, University of Electronic Science and Technology of China, between December 2017 and January 2022 were analyzed. Basic patient information, Bismuth–Corlette type, AJCC staging, postoperative complications, pathological findings, and follow-up results were evaluated.

**Results:**

Sixteen patients underwent LS and 9 underwent OS for HC. According to the preoperative imaging data, there were four cases of Bismuth type IIIa, eight of type IIIb, and four of type IV in the LS group and two of type IIIa, four of type IIIb, and three of type IV in the OS group (*P*>0.05). There were no significant differences in age, sex, ASA score, comorbidity, preoperative percutaneous transhepatic biliary drainage rate, history of abdominal surgery, or preoperative laboratory tests between the two groups (*P*>0.05). Although the mean operative time and mean intraoperative blood loss were higher in the LS group than in OS group, the differences were not statistically significant (*P*=0.121 and *P*=0.115, respectively). Four patients (25%) in the LS group and two (22.2%) in the OS group experienced postoperative complications (*P*>0.05). No significant differences were observed in other surgical outcomes and pathologic findings between the two groups. Regarding the tumor recurrence rate, there was no difference between the groups (*P*>0.05) during the follow-up period (23.9 ± 13.3 months vs. 17.8 ± 12.3 months, *P*=0.240).

**Conclusion:**

Laparoscopic radical resection of Bismuth types III and IV HC remains challenging, and extremely delicate surgical skills are required when performing extended hemihepatectomy followed by complex bilioenteric reconstructions. However, this procedure is generally safe and feasible for hepatobiliary surgeons with extensive laparoscopy experience.

## 1 Introduction

Cholangiocarcinoma (CCA) is an aggressive tumor of the biliary tract that accounts for approximately 10% of all hepatobiliary tumors and 3% of all gastrointestinal malignancies (1). It arises from biliary epithelial cells and their progenitor cells and can develop at various levels of the intrahepatic and extrahepatic biliary tree. CCA is divided into intrahepatic, hilar (HC; or Klatskin tumor), and extrahepatic types according to its anatomical location. Originally described by Klatskin in 1965, HC occurs more frequently than the other CCA types, accounting for two-thirds of all CCA cases (2). The most common clinical symptoms of HC are obstructive jaundice with or without pain in the abdomen, weight loss, pruritis, and discomfort (3). Owing to a lack of early symptoms, only one in five patients with HC are estimated to be eligible for surgery at the time of presentation (4).

The prognosis of HC is poor, particularly when lymph node metastasis or vascular invasion occurs. Radical surgical resection is the only means of curing the disease and prolonging patient survival. Considerable technological advances have been made recently in hepatobiliary surgery. However, HC surgery represents a significant challenge, particularly when performed laparoscopically, despite the development of various strategies for achieving R0 resection, including standard or extended hepatectomy combined with cholecystectomy, removal of the extrahepatic biliary tract, lymphadenectomy, and bilioenteric restoration. The overall aim of the surgery is R0 resection, which is necessary for improved survival (5). Owing to the continuous efforts of hepatobiliary surgeons, there have been significant advances in minimally invasive techniques which are now used for treating hepatobiliary malignancies. With increasing experience in laparoscopic hepatectomy and pancreaticoduodenectomy, the application of laparoscopic resection has expanded to HC, increasing the safety and feasibility of complete resection of tumors with R0 resection in specifically selected cases. Radical resection of Bismuth types III and IV HC under laparoscopy is a more challenging procedure than radical resection of Bismuth types I and II HC and has been rarely reported worldwide (6). Here, we describe the experience with laparoscopic surgery (LS) for HC at our center with the aim to analyze the feasibility and safety of this surgery for the treatment of Bismuth type III and IV HC.

## 2 Materials and methods

### 2.1 Patients and patient data

The data of consecutive patients who underwent radical resection of Bismuth type III and IV HC were retrospectively collected from a single-center database. These patients had been admitted to the Department of Hepatobiliary Surgery, Mianyang Central Hospital, University of Electronic Science and Technology of China between December 2017 and January 2022. Patients with intrahepatic CCA or gallbladder cancer were excluded. At the time of enrollment, none of the patients showed evidence of metastatic disease on imaging that was considered non-radically resectable. The inclusion criteria were that the tumor had not invaded the trunks or branches of the portal vein or hepatic artery, and the absence of extensive intrahepatic and distant metastases were absent. All patients provided informed consent, and the cases were reviewed and approved by the Medical Ethics Committee of Mianyang Central Hospital, School of Medicine, University of Electronic Science and Technology, China.

### 2.2 Preoperative evaluation and management

**Figure 1:**
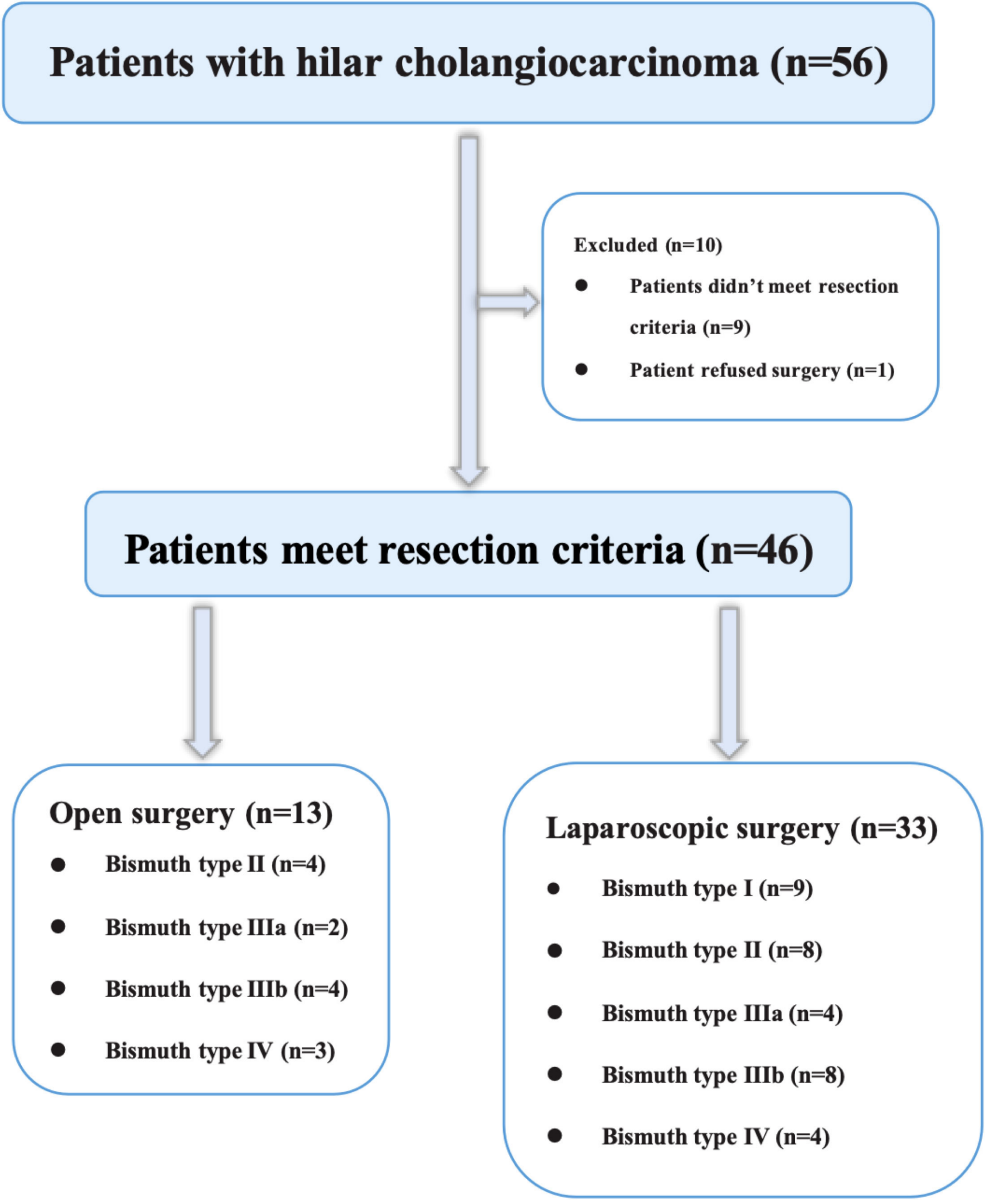
Flow chart of the study design and participant eligibility.

The success of the procedure depends on tumor resectability, the residual liver volume, and general condition of the patient. At our center, we typically choose cross-sectional contrast-enhanced computed tomography (CT) and/or magnetic resonance imaging (MRI) to assess the resectability of HC. In brief, the criteria used to assess unresectability of Bismuth type III and IV HC were (a) tumor invasion of the main trunks or bilateral branches of either the portal vein or hepatic artery, (b) extensive intrahepatic and distant metastases, (c) insufficient volume of the remaining liver, and (d) inability to tolerate general anesthesia. During our study period, nine patients with Bismuth type III or IV HC underwent open surgery (OS) either at the request of the patient or because imaging data showed possible hepatic vessels invasion (**Figure 1**).

The liver volume was assessed in all patients using enhanced CT and three-dimensional reconstruction before surgery, especially in patients who were likely to undergo extended hepatectomy. Assessing residual liver function can minimize the likelihood of liver insufficiency or failure following surgery. During radical tumor resection, a minimal residual volume of 30% should be present in patients with normal function and reasonably good physical condition, while this should be increased to 40% in cases with severe obstructive jaundice or cirrhosis.

Bile draining is not always necessary in the presence of jaundice, and the decision to perform bile drainage preoperatively depends on whether the benefits outweigh the risk of perioperative complications. The indications for bile drainage at our center are (a) patients with total serum bilirubin >200 μmol/L who require combined extensive liver resection (>60% of the lobes removed), (b) presence of cholangitis, (c) patients who are at a high nutritional risk, and (d) those who require selective portal vein embolization. Percutaneous transhepatic cholangial drainage (PTCD), endoscopic nasobiliary drainage (ENBD), and endoscopic retrograde biliary drainage (ERBD) are the most commonly used preoperative bile drainage methods; however, the specific method of bile drainage mainly depends on the experience of each medical center. We prefer using PTCD to ENBD or ERBD in our center because the inflammation around the biliary ducts or pancreatitis due to ENBD or ERBD can complicate subsequent surgery and lymph node dissection. Moreover, ENBD and ERBD are relatively easier to apply for treating Bismuth types I and II HC; however, for Bismuth types III and IV HC, ENBD and ERBD typically cannot completely resolve obstructive jaundice. Our center prefers unilateral drainage of the reserved liver lobe to effectively reduce jaundice while increasing the functional compensation of the reserved liver lobe. Surgery is usually deferred for ≥2 weeks to allow for adequate recovery of liver function.

### 2.3 Surgical procedure

#### 2.3.1 Laparoscopic surgery

##### 2.3.1.1 Patient positioning and trocar distribution

**Figure 2:**
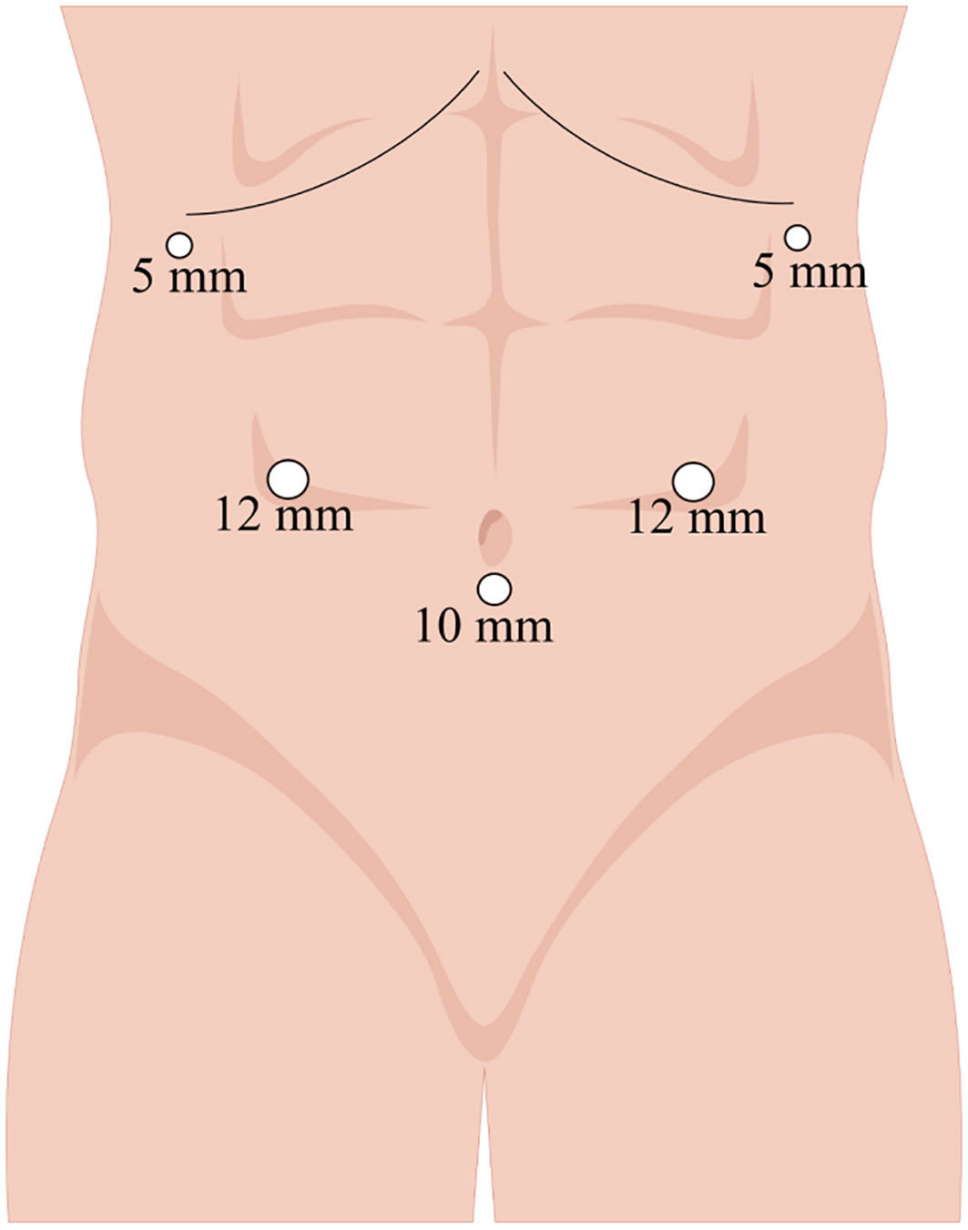
Trocar placement on the abdomen.

Anesthetized patients were positioned in a supine reverse Trendelenburg position with their legs apart. The surgeon was situated on the right side of the patient, the assistant on the left, and the camera operator between the patient’s legs. After establishment of pneumoperitoneum by puncture below the umbilicus, pressure was maintained at <14 mmHg. **Figure 2** shows the trocar distribution, with placement of the camera trocar 10 mm below the umbilicus, two 12-mm trocars 2 cm above the umbilicus on the midclavicular lines (right and left, respectively), and two 5-mm trocars on the right and left anterior axillary lines 1 cm below the costal margin.

##### 2.3.1.2 Abdominal exploration and specimen resection

The abdomen was completely explored to rule out the presence of metastases. Approximately 27–45% of potentially resectable tumors are considered unresectable at laparoscopic exploration (7, 8). The gallbladder was separated and resected from the bed. Subsequently, dissection of the bile duct and liver plate was performed to expose the secondary bile ducts in the anticipated liver lobe, which helps confirm the exact extent of tumor infiltration and determine the presence of invasion into the hepatic vessels and their branches. It is not recommended to perform hepatectomy unless the hepatic vessels on both sides are completely exposed, especially for Bismuth type IV HC. After selecting the surgical procedure, the Kocher maneuver was used for mobilization of the pancreatic and duodenal heads, and the para-aortic lymph nodes (No. 16) were routinely dissected for frozen section biopsy to avoid potential metastases. Resection of the hepatoduodenal ligament was performed along the superior boundary of the duodenum, together with lymph nodes 1, 3, 5, and 7. Next, the head of the pancreas was shifted to the left, followed by removal of the number 13 lymph nodes and number 8 lymph nodes on the common hepatic artery. The distal part of the common bile duct at the superior border of the pancreatic head was removed and frozen for pathological analysis. If tumor cells were detected microscopically at the distal margin, combined laparoscopic pancreaticoduodenectomy was performed. This was followed by lifting and sealing of the proximal stump of the common bile duct and, after dissecting the common hepatic artery and celiac trunk, the number 9 lymph nodes were removed. The number 12 lymph nodes were resected after exposure and skeletonization of the portal and arterial structures at the hepatic hilum.

It is possible to perform the Pringle maneuver safely for 15 min with several repeats during liver resection. Therefore, liver resection was conducted using an alternate Pringle maneuver with reduced pressure on the central veins. The surgical protocol for liver resection depends mainly on the Bismuth type. Because the caudate lobe bile ducts often converge into the left or right hepatic ducts or their confluence, they are easily invaded by the tumor; therefore, the caudate lobe was removed at the same time at our center. If the tumor was Bismuth type IIIa, right hemihepatectomy was performed; Bismuth type IIIb, left hemihepatectomy was performed; and Bismuth type IV, right or left trisectionectomy or central hepatectomy was performed. We dissected the normal bile duct at least 1 cm above the tumor (the cut edges are routinely sent for frozen pathology) and then removed the tumor completely. Specimen removal was undertaken via a 5-cm longitudinal incision in an upper abdomen or a transverse incision of similar length in the superior symphysis pubis.

After removing the liver specimen, hepaticojejunostomy was undertaken using continuous sutures along the anterior and posterior walls of the anastomoses and Roux-en-Y reconstruction was conducted in an ante-colonic manner. The surgical field was flushed with saline, and bile leakage and bleeding were carefully monitored. At the end of the surgery, one drainage tube was positioned around the hepaticojejunostomy, with another tube beneath the liver.

#### 2.3.2 Open surgery

The surgery for OS was performed using a reverse “L” incision (approximately 16–20 cm), similar to the procedures of LS, and will not be discussed further.

### 2.4 Data analysis

Perioperative characteristics of the patients were collected, including the findings of laboratory examinations, Bismuth–Corlette classification, operative time, intraoperative blood loss, transfusion requirement, type of hepatectomy, surgical radicality, tumor size, lymph node dissection, postoperative hospital stay, complications, pathology, and American Joint Committee on Cancer stage. Follow-up evaluations were performed during clinic visits or telephone calls for the assessment of tumor recurrence and prognosis.

Continuous data are expressed as the mean ± standard deviation (SD) or median (range) and were compared using Student’s t-test or Mann–Whitney U test, respectively. Categorical data are expressed as numbers and percentages, and Chi-square tests or Fisher’s exact tests were used to compare these data. In all analyses, *P*<0.05 was considered statistically significant. All analyses were performed using SPSS (version 23.0; IBM Corp., Armonk, NY, USA).

## 3 Results

### 3.1 Characteristics of patients

The final analysis included 16 patients in the LS group and 9 in OS group with Bismuth type III and IV HC (**Figure 1**). The preoperative features of the patients are shown in **Table 1**. The mean age was 66.50 ± 5.55 years in the LS group and 65.78 ± 4.35 years in OS group (*P*=0.748). The primary preoperative clinical manifestations included mild abdominal pain, progressive exacerbation of jaundice, general fatigue, nausea, and anorexia. Seven patients (43.75%) in the LS group and seven (77.8%) in OS group had comorbidities (*P*=0.210), while nine (56.25%) in LS group and four (44.4%) in OS group (*P*=0.688) had undergone PTCD before surgery for severe obstructive jaundice. The laboratory results of LS group were as follows: white blood cell count (6.10 ± 2.44) ×10^9^/L, red blood cell count (3.76 ± 0.63) ×10^12^/L, platelet count (184 ± 68.2) ×10^9^/L, hemoglobin (112 ± 16.8) g/L, prothrombin time (11.6 ± 1.22) s, total bilirubin (155.3 ± 103.3) μmol/L, direct bilirubin (136.1 ± 82.18) μmol/L, and CA-199 (880.0 ± 857.0) U/mL, which were not significantly different from those in the OS group (*P*>0.05). According to the Child–Pugh classification criteria, all patients (100%) had liver function of Child–Pugh clasps A preoperatively. In the LS group, Bismuth type IIIa HC was present in four (25%) cases, type IIIb HC in eight (50%), and type IV HC in four (25%), whereas in the OS group, Bismuth type IIIa HC was seen in two (22.2%), type IIIb HC in four (44.4%), and type IV HC in three (33.3%) cases (*P*>0.05). Overall, there were no significant differences between the two groups with respect to the age, ASA score, comorbidity, preoperative percutaneous transhepatic biliary drainage rate, history of abdominal surgery, or preoperative laboratory tests (*P*>0.05).

**Table 1:** Patients’ preoperative characteristics.

Characteristics	LS group	OS group	*P* value
Gender, n (%) Male Female	10 (62.5) 6 (37.5)	5 (55.6) 4 (44.4)	0.734
Age, year	66.50 ± 5.55	65.78 ± 4.35	0.748
Comorbidity, n (%) Hypertension Diabetes Coronary atherosclerotic heart disease Hyperlipidemia	2 (12.5) 3 (17.75) 1 (6.25) 1 (6.25)	3 (33.3) 1 (11.1) 3 (33.3) 0 (0.0)	0.210
Biliary drainage (PTCD), n (%)	9 (56.25)	4 (44.4)	0.688
Previous abdominal surgery, n (%)	3 (18.75)	2 (22.2)	1.000
ASA score, n (%) I II	12 (75.0) 4 (25.0)	7 (77.8) 2 (22.2)	0.876
Laboratory tests WBC (10^9/L), median (IQR) RBC (10^12/L), median (IQR) PLT (10^9/L), median (IQR) HB (g/L), median (IQR) PT (s), median (IQR) TBIL (μmol/L), median (IQR) DBIL (μmol/L), median (IQR) ALT(U/L), median (IQR) AST(U/L), median (IQR) ALB(g/L), median (IQR) CA-199 (U/mL), median (IQR) CA-125 (U/mL), median (IQR) CEA (ng/ml), median (IQR) AFP (U/ml), median (IQR)	6.10 ± 2.44 3.76 ± 0.63 184 ± 68.2 112 ± 16.8 11.6 ± 1.22 155.3 ± 103.3 136.1 ± 82.18 173.6 ± 129.7 129.1 ± 77.65 40.12 ± 5.93 880.0 ± 857.0 17.2 ± 8.42 2.85 ± 1.23 3.13 ± 1.45	6.35 ± 1.53 3.39 ± 0.71 177 ± 51.2 103 ± 16.4 11.2 ± 0.96 151.8 ± 76.79 111.5 ± 74.65 181.1 ± 82.03 129.3 ± 68.75 42.47 ± 10.40 498.0 ± 493.0 18.2 ± 7.16 2.57 ± 0.61 3.13 ± 0.72	>0.9999 0.257 0.856 0.211 0.606 0.749 0.452 0.419 0.966 0.760 0.595 0.572 0.759 0.770
Preoperative Bismuth type, n (%) IIIa IIIb IV	4 (25.0) 8 (50.0) 4 (25.0)	2 (22.2) 4 (44.4) 3 (33.3)	1.000
Child-Pugh A, n (%)	16 (100.0)	9 (100.0)	1.000
T stage, n (%) T1 T2 T3	9 (56.25) 6 (37.5) 1 (6.25)	3 (33.3) 4 (44.4) 2 (22.2)	0.458

LS, laparoscopic surgeryl; OS, open surgery; PTCD, percutaneous transhepatic cholangial drainage; ASA, American Society of Anesthesiologists; IQR, interquartile spacing; WBC, white blood cells; RBC, red blood cells; PLT, platelets; HB, hemoglobin; PT, prothrombin time; TBIL, total bilirubin; DBIL, direct bilirubin; ALT, alanine aminotransferase; AST, aspartate aminotransferase; ALB, albumin; CEA, carcinoembryonic antigen; AFP, alpha fetoprotein.

### 3.2 Intraoperative parameters

**Figure 3:**
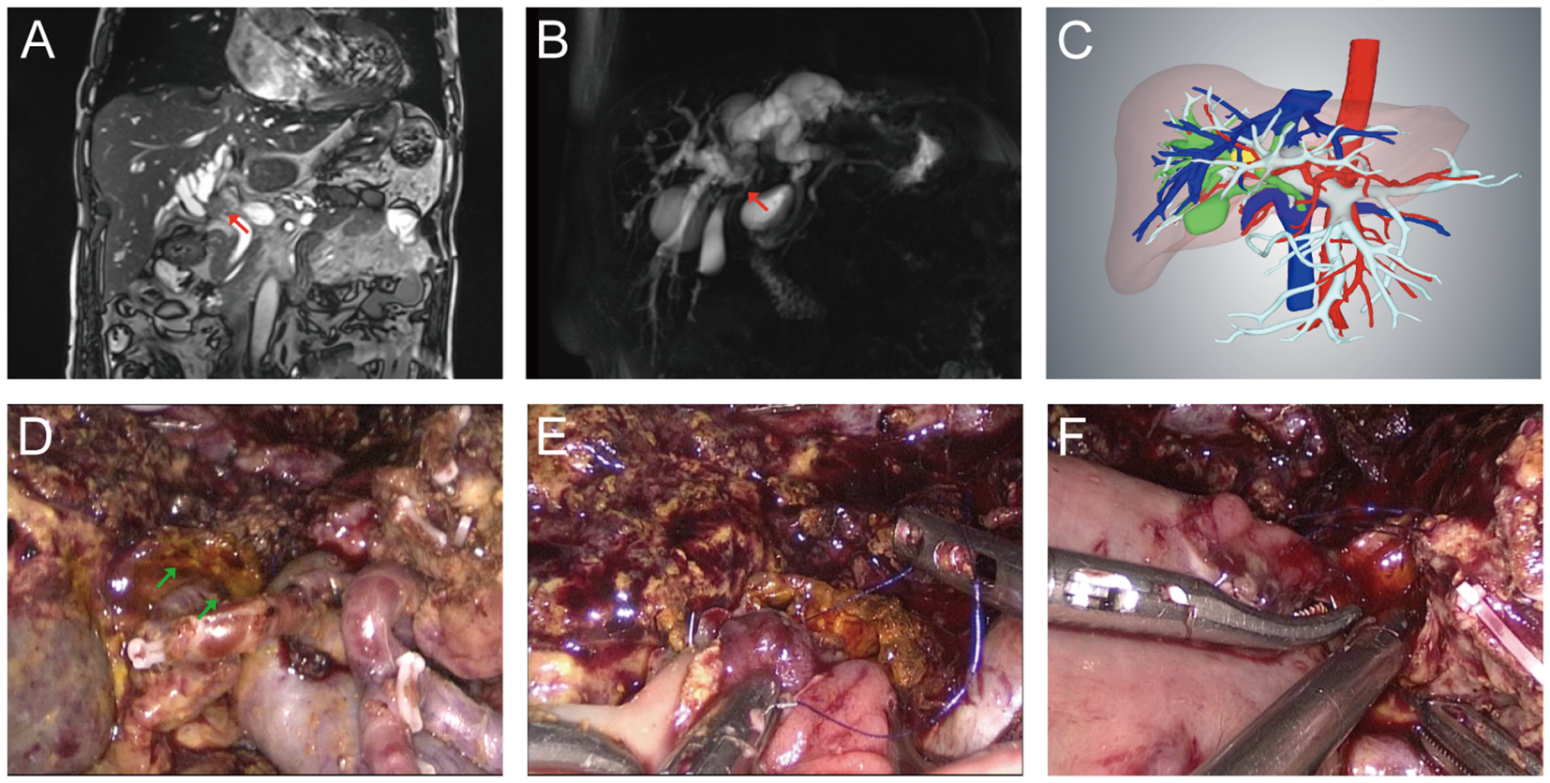
Typical Bismuth type IV HC patient who received laparoscopic radical resection. **(A)** Preoperative MRI image. The red arrow indicated where the tumor of bile duct was located. **(B)** Preoperative MRCP image. The red arrow indicated where the tumor was located. **(C)** Preoperative 3-D reconstruction model of the Bismuth type IV HC patient. The yellow area indicated by the red arrow was the tumor. **(D)** The hepatic hilum after central hepatectomy and extended lymphadenectomy. Skeletonized portal vein and hepatic artery were visible and the green arrow indicated the bile duct opening after central hepatectomy. **(E)** Biliary-intestinal anastomosis of the right hepatic duct section. **(F)** Biliary-intestinal anastomosis of the left hepatic duct section.

In all patients of the LS group, laparoscopic radical surgery was successfully performed without convertion to OS. The intraoperative parameters of the LS and OS groups are provided in **Table 2**. Although the mean operative time and mean intraoperative blood loss were higher in the LS group than in OS group, the differences were not statistically significant (601.0 ± 183.0 min vs. 472.0 ± 70.6 min, *P*=0.121; 559.0 ± 567.0 mL vs. 189.0 ± 108.0 mL, *P*=0.115). In terms of blood transfusions, the red blood cell and plasma requirements were 0 U (0–3.75) and 0 ml (0–400) in the LS group, and 0 U (0–3.25) and 0 mL (0–350) for red blood cells and plasma, respectively, in the OS group (*P*>0.05). In LS group, laparoscopic left hemihepatectomy was conducted in 8 patients, with laparoscopic right hemihepatectomy in 4, laparoscopic left trisectionectomy in 2, and laparoscopic central hepatectomy in 2 (**Figure 3**), and R0 margins were achieved in 14 patients. Surgical specimens from one type IV HC patient and one type IIIb HC patient in LS group suggested that a few tumor cells were present in the hepatic duct section because their tumors reached the tertiary bile ducts and could not be further resected, while the R0 resection rate was 55.6% in the OS group (*P*=0.142). The Bismuth classification was not altered in any of the patients following surgery. However, there was a change in T stage in one type IIIb patient in LS group due to tumor invasion of the lateral portal vein branch, which was not visible on preoperative CT or MRI.

**Table 2:** Patients’intraoperative parameters.

Parameters	LS group	OS group	*P* Value
Operating time (min), mean (SD)	601.0 ± 183.0	472.0 ± 70.6	0.121
Estimated blood loss (mL), mean (SD)	559.0 ± 567.0	189.0 ± 108.0	0.115
Transfusion requirement Red cell (U), median (IQR) Plasma (ml), median (IQR)	0 (0, 3.75) 0 (0, 400)	0 (0, 3.25) 0 (0, 350)	1.000
Type of hepatectomy, n (%) Left hemihepatectomy Right hemihepatectomy Left trisectionectomy Central hepatectomy	8 (50.0) 4 (25.0) 2 (12.5) 2 (12.5)	4 (44.4) 2 (22.2) 1 (11.1) 2 (22.2)	0.930
Surgical radicality, n (%) R0 R1	14 (87.5) 2 (12.5)	5 (55.6) 4 (44.4)	0.142
Bismuth type, n (%) IIIa IIIb IV	4 (25.0) 8 (50.0) 4 (25.0)	2 (22.2) 4 (44.4) 3 (33.3)	1.000
Tumor size (cm), mean (SD)	2.44 ± 0.67	2.63 ± 0.81	0.410
Lymph node dissection, mean (SD)	7.88 ± 3.53	5.22 ± 1.09	0.028
T stage, n (%) T1 T2 T3	9 (56.25) 5 (31.25) 2 (12.5)	4 (44.4) 3 (33.3) 2 (22.2)	0.859

### 3.3 Postoperative and pathological outcomes

Patient outcomes, both postoperative and pathological, are shown in **Table 3**. The duration of postoperative hospital stay was 35.2 ± 13.8 days in the LS group and 35.2 ± 9.9 days in OS group (*P*=0.770), including that of patients who experienced complications. Complications in the LS group occurred in four cases (25.0%): bile leakage, abdominal infection, hypoproteinemia, and intra-abdominal bleeding, each occurring in one patient. The patient with bile leakage recovered after intensive drainage and nutritional support. After undergoing intensive drainage and anti-infection treatment, the patient with abdominal infection also recovered. The patient who developed hypoproteinemia recovered after infusion of human albumin. The etiology of the patient’s intra-abdominal bleeding was considered to be a ruptured gastroduodenal artery, and the patient recovered after interventional treatment. Two complications (22.2%) occurred in the OS group, bile leakage and abdominal infection (*P*>0.05). According to the Clavien-Dindo classification, two patients in the LS group (12.5%) experienced grade I–II complications, and two (12.5%) experienced grade III complications. In the OS group, one patient (11.1%) experienced grade I–II complications, and one (11.1%) experienced grade III complications (*P*>0.05). According to the 8th edition of AJCC staging system, stage I tumors were present in seven patients, stage II in five, stage IIIB in one, stage IIIC in tw, and stage IVA in one in the LS group. Pathological results showed bile duct adenocarcinoma and invasive intracapsular papillary carcinoma in 15 (93.75%) and 1 (6.25%) patients, respectively. During the follow-up period, seven patients (43.75%) in the LS group and three (33.3%) in OS group were alive without showing any symptoms of recurrence (*P*>0.05). Nine patients (56.25%) in the LS group experienced postoperative tumor recurrence; of these, five (31.25%) had peritoneal recurrence, two (12.5%) liver recurrence, and two (12.5%) multiple metastases in the hilar region and lymph nodes. In contrast, five patients (55.5%) in the OS group experienced postoperative tumor recurrence; of these, one (11.1%) had peritoneal recurrence, and four (44.4%) had liver recurrence (*P*>0.05). Eight patients (50.0%) in the LS group and four (44.4%) in OS group died during follow-up (*P*>0.05), and the causes of death were tumor recurrence and metastasis.

**Table 3:** Patients’ postoperative parameters and pathological results.

Parameters	LS group	OS group	*P* Value
Postoperative hospital stay (days)	35.2 ± 13.8	35.2 ± 9.9	0.770
Complications, n (%) Bile leakage Abdominal infection Hypoproteinemia Intra-abdominal bleeding	4 (25.0) 1 (6.25) 1 (6.25) 1 (6.25) 1 (6.25)	2 (22.2) 1 (11.1) 1 (11.1) 0 (0.0) 0 (0.0)	1.000
Clavien–Dindo grading system I:II:III:IV:V	1:1:2:0:0	0:1:1:0:0	1.000
90-day mortality, n (%)	0 (0.0)	0 (0.0)	1.000
Pathology, n (%) Low-moderate differentiated bile duct adenocarcinoma Moderately differentiated bile duct adenocarcinoma High-moderate differentiated bile duct adenocarcinoma Invasive intracapsular papillary carcinoma	5 (31.25) 9 (56.25) 1 (6.25) 1 (6.25)	2 (22.2) 5 (55.6) 2 (22.2) 0 (0.0)	0.805
AJCC stage, n (%) I II IIIB IIIC IVA	7 (43.75) 5 (31.25) 1 (6.25) 2 (12.5) 1 (6.25)	4 (44.4) 3 (33.3) 1 (11.1) 0 (0.0) 1 (11.1)	1.000
Follow-up, median (months)	23.9 ± 13.3	17.8 ± 12.3	0.240
Recurrence, n (%)	9 (56.25)	5 (55.6)	1.000
Death, n (%)	8 (50.0)	4 (44.4)	1.000

## 4 Discussion

Currently, the indications for LS for Bismuth types III and IV HC are not well-defined; therefore, the surgical methods and indications for patients with HC must be determined carefully. In our study, we selected highly resectable tumors without vascular invasion or liver lobe atrophy seen in preoperative imaging. The main objective of preoperative imaging studies is not only to confirm the presence of tumors but also evaluate the degree of involvement of the bile duct, as well as vascular involvement and the presence of distant metastases, which are essential for surgical planning. In our experience, performing all imaging procedures before biliary drainage is important because the accuracy of the assessment can be significantly impaired, especially in cases where the tumor has invaded the secondary biliary ducts. CT and MRI are the most widely used forms of imaging for the evaluation of HC (9). CT can better define local tumor extension, vascular invasion, and metastatic disease (10). A Dutch research group reported an accuracy of 86% in determining tumor ductal extension on CT, whereas the sensitivity and specificity were 89% and 92%, respectively, for evaluation of the involvement of the portal vein, 83% and 93%, respectively, the hepatic artery, and 61% and 88%, respectively, for lymph nodes (11). Moreover, multirow CT (high resolution) has an increased ability to predict biliary tumor spread in the HC ducts, especially when the bile ducts are dilated (12).

MRI with MR cholangiopancreatography and diffusion-weighted imaging have additional value in assessing the biliary extent of the tumor, and MR cholangiopancreatography has the best sensitivity and accuracy in identifying HC extensions (92% and 76%, respectively) (13). Positron emission tomography–CT should not be routinely used in diagnostic workups because of its lower sensitivity and specificity in the detection of tumor invasion; however, it plays a substantial role in tumor characterization and metastasis detection (14). Positron emission tomography–CT may be a reasonable choice in some cases, with a 10% yield of occult metastatic disease (15). Direct cholangiography, including endoscopic retrograde cholangiopancreatography and percutaneous transhepatic cholangiography, can offer evidence on the structure of the biliary tree. Endoscopic retrograde cholangiopancreatography with biliary brushing is used in the diagnostic workup of HC. However, the sensitivity of cytology alone is as low as 5–40%, although the specificity is 100% (16). Despite the availability of advanced technology for reaching an accurate preoperative diagnosis, accurate staging and classification of HC is not always possible, and several patients have been found to have unresectable locally advanced tumors or occult metastases at the time of surgical exploration. It has been estimated that only 80% of cases have preoperative diagnoses that are identical to the pathological diagnosis (17). In our study, one patient with Bismuth type IIIb showed invasion of the branch to the lateral portal vein that was not visible on preoperative imaging. Although preoperative imaging is necessary for the assessment of tumor resectability and decision on the surgical approach, imaging limitations together with varying interpretations of the findings can result in inconsistency between the intraoperative observations and preoperative assessment; this should be considered for a successful HC surgery.

Preoperative bile drainage in surgical candidates for HC has been advocated for as well as criticized, and the indications and means of decompression remain controversial. Obstructive jaundice has been associated with increased postoperative mortality, and patients with jaundice have impaired functioning of the intestinal barrier, facilitating the transfer of bacteria. Decompression has been confirmed to lower the likelihood of liver failure and death following surgery (18, 19). Several studies have highlighted the high incidence of complications following liver resection in cases with jaundice, indicating that preoperative biliary drainage is beneficial in these cases (20–22). However, some studies have suggested that manipulation of the biliary tract is associated with complications such as hemorrhage and sepsis, potentially increasing postoperative morbidity and mortality (23, 24). On the basis of these assumptions, at our center, we recommend the use of biliary drainage prior to surgery in patients who are likely to have only small amounts of residual liver or who will undergo preoperative portal vein embolization, and preoperative bile drainage is mandatory in patients who present with cholangitis. PTCD and ENBD are the most commonly used bile drainage methods. Compared with ENBD, PTCD results in fewer conversions and a lower incidence of pancreatitis and cholangitis (25), although PTCD has the risk of seeding metastases (26). However, the specific choice of the preoperative bile drainage method depends mainly on the experience of each medical center.

Surgery is the only curative option for HC and the main goal is to achieve R0 resection. The main recognized problems with laparoscopic resection of the HC are the inability to palpate the hilum and the technical challenges associated with hepaticojejunostomy, which are the reasons why some researchers oppose LS. In our experience, during LS for HC, the hilus should first be dissected using direct vision and its resectability evaluated before conducting resection. Specifically, the gallbladder was detached from the liver bed before dissection of the liver plate along the wall of the bile duct, thus exposing the bifurcation and secondary bile ducts, confirming the precise extent of the tumor and determining the presence or absence of tumor invasion of the hepatic vessels. Therefore, we can technically assess the resectability of the hilum and perform laparoscopic radical surgery without direct palpation. For most HC cases, extended hemihepatectomy, including en bloc resection of the extrahepatic bile duct, portal lymphadenectomy, and Roux-en-Y hepaticojejunostomy must be performed. The specific form of the hepatectomy required depends on the degree of biliary invasion: right hemihepatectomy is required for type IIIa tumors, left hemihepatectomy for type IIIb tumors, and right or left trisectionectomy or central hepatectomy (segments 1, 4B, and 5) for type IV tumors. Due to the characteristic infiltration involved in HC growth, as well as venous drainage between the liver and hilar bile duct, the tumor may penetrate the caudate lobe either by direct infiltration or through a tumor embolus, often necessitating the complete resection of the caudate lobe during LS for HC (27, 28). Bhutiani et al. (29) confirmed that such resection of the caudate lobe increased the likelihood of R0 resection, with no difference in morbidity or mortality. On comparing between the American and Japanese cohorts, the caudate resection rates were 8% and 89%, respectively, with favorable survival rates (30). The American Extrahepatic Biliary Malignancy Consortium also reported a reduced rate of R1 or R2 margins with caudate resection; however, this was not associated with a difference in recurrence-free or overall survival compared with that in patients who did not undergo caudate resection (29). At our center, the caudate lobe is routinely resected to increase R0 resection.

The presence of lymph node metastases adversely affects HC prognosis, with 5-year survival rates of only 0–25% (31). The regional and para-aortic nodes are most commonly implicated in HC. Regional nodes, as defined by the International Union against Cancer, are those located in the cystic duct, hilar, periportal, periduodenal, celiac, peripancreatic, and pericholedochal regions, as well as those in the superior mesentery (32). Yuichi et al. (33) reported that the most commonly affected were pericholedochal (42.7%), periportal (30.9%), common hepatic (27.3%), and posterior pancreaticoduodenal (14.5%) nodes. These authors observed 3- and 5-year survival rates of 55.4% and 30.5%, respectively, in 52 patients with no node involvement, 31.8% and 14.7%, respectively, in 39 with regional node involvement, and 12.3% and 12.3%, respectively, in 19 with para-aortic node involvement. Bagante et al. (34) suggested that extensive lymphadenectomy, together with removal of the para-aortic nodes, may extend patient survival. LS does not allow palpation for the identification of affect lymph nodes. Therefore, our center routinely recommends the removal of all potentially affected lymph nodes, including the regional and para-aortic nodes, which may be beneficial in prolonging patient survival.

In contrast to OS, intraoperative frozen pathological sections are important to ensure surgical radicality and specimens should be collected during dissection. The surgeon should have an overall view of the degree of tumor invasion from the preoperative imaging findings. In cases where type I or II HC is suspected, the achievement of R0 resection depends more on the lower border of the distal common bile duct than the upper. Pancreaticoduodenectomy should be performed in cases of positivity of the distal margin superior to the duodenum and, in cases with suspected type III or IV HC, negativity of the upper margin of the bile duct should be confirmed pathologically.

Gumbs et al. (35) attempted to perform laparoscopic radical surgery for HC in 2013, and currently, some studies aimed to compare the short-term and long-term outcomes between laparoscopic versus OS for HC (36–38). However, their findings remain controversial. For example, Ma et al. (36) evaluated the outcomes of LS and compared them to those of OS. Their results suggested that LS was equivalent to OS in terms of short-term outcomes but poorer in terms of long-term outcomes. In contrast, He et al. (38) and Qin et al. (39) reported that the short-term and long-term outcomes were comparable. In our study, there were no advantages in most intraoperative outcomes and postoperative outcomes in the LS group compared with OS group. More, our results showed that there was no statistically significant difference in short-term outcomes (including tumor recurrence rate) between the LS and OS groups, which was similar to the results in the study by Ma et al. (36). The long-term outcomes of LS need to be further confirmed in larger cohorts and higher quality clinical studies. Combined with the treatment experience of our center, we conclude that LS for Bismuth types III and IV HC has the following advantages compared with OS: (a) hepatoportal space is narrow, the field of view is narrow, difficult to reveal, whereas laparoscopic observation angle is flexible and has the effect of magnification; therefore, it can allow more satisfactory surgical field and operation space; (b) the anterior space of the posterior hepatic vena cava is an important site for surgical operation of hepatoportal cholangiocarcinoma. Compared with OS, laparoscopic caudate lobectomy can better show the anterior space of the posterior hepatic vena cava, and it is easier to isolate the caudate vein, the right posterior inferior hepatic vein and manage the third hepatic hilar under direct vision, (c) laparoscopic lymph node dissection can be achieved by combining anterior and posterior approaches to achieve the full extent of open surgical dissection, (d) LS involves smaller incision, which helps to minimize postoperative incision-related complications.

To improve the prognosis in patients, several studies have attempted to utilize radiomics and artificial intelligence (AI) (including machine learning) for early diagnosis and postoperative surveillance of CCA (40–42). For example, Yang et al. (43) developed an MRI-based AI model for diagnosing patients with CCA as well as evaluating the extent and severity of lymph node metastasis. In their study, after training the model on 100 patients, this AI model could differentiate between those at high and low risk for CCA and between metastatic and non-metastatic lymph nodes, with AUCs of 80% and 90% for the test cohorts, respectively (43). Nakai et al. (44) developed a convolutional neural network (CNN) model that combines CT and serum tumor markers (including CEA and CA199), and this CNN model outperformed human radiologists in CCA diagnose (*P*=0.04). In addition, Song et al. (45) developed a preoperative model for predicting the risk of early postoperative recurrence of iCCA using an AI-based CT radiomics approach. The sensitivity of this machine learning model could reach 94.6% on average. Similarly, another study suggested that CNN-based machine leaning of cholangioscopy images might benefit patients with malignant biliary strictures and CCA (46). However, although radiomics and AI exhibit great potential for the diagnosis, treatment, and prognosis of CCA, there are still some issues that should be considered in the future, such as ethical concerns and the acceptance of these technologies by patients.

### 4.1 Limitations

Although our study demonstrates the safety and feasibility of total laparoscopic radical resection for HC, it had some limitations. First, the sample size was small (16 patients), accompanied by long hospitalization (35.18 ± 13.77 days). Four patients (25%) experienced complications following surgery (bile leakage, abdominal infection, hypoproteinemia, and intra-abdominal bleeding in one patient each), and nine (56.25%) underwent preoperative PTCD, resulting in a delay of surgery by ≥2 weeks, which may have contributed to the overall hospital stay. Another limitation is that routine extensive lymphadenectomy in cases with Bismuth types III and IV HC is not universally accepted, and supporting data are currently insufficient. At our center, we routinely remove all potentially involved lymph nodes, including para-aortic nodes, as this may prolong patient survival.

### 4.2 Conclusions

In LS for Bismuth types III and IV HC, the hepatic hilum should be dissected under direct vision to allow the assessment of resectability before undertaking resection since direct palpation is not possible. The collection of pathological specimens during surgery is important to ensure the success of the resection. During LS for HC, the lymph nodes infiltrated by the tumor cannot be identified by direct palpation and thus routine extensive lymphadenectomy should be conducted because this may benefit patient outcomes. These techniques enhance the radicality of laparoscopic resection of Bismuth types III and IV HC. Despite the need for precise and delicate surgical skills when performing (extended) hemihepatectomy followed by complex bilioenteric reconstructions, laparoscopic radical resection of Bismuth types III and IV HC is both safe and feasible for hepatobiliary surgeons with laparoscopy experience.

## Data availability statement

The original contributions presented in the study are included in the article/supplementary material, further inquiries can be directed to the corresponding author/s.

## Ethics statement

The studies involving humans were approved by the Medical Ethics Committee of Mianyang Central Hospital, School of Medicine, University of Electronic Science and Technology, China. The studies were conducted in accordance with the local legislation and institutional requirements. Written informed consent for participation in this study was provided by the participants’ legal guardians/next of kin. Written informed consent was obtained from the individual(s) for the publication of any potentially identifiable images or data included in this article.

## Author contributions

JW: Conceptualization, Methodology, Project administration, Software, Visualization, Writing – original draft. YX: Conceptualization, Methodology, Validation, Writing – review & editing. YC: Methodology, Project administration, Writing – review & editing. XZ: Conceptualization, Supervision, Validation, Writing – original draft. HL: Data curation, Software, Writing – review & editing. XC: Methodology, Resources, Writing – original draft. MS: Funding acquisition, Writing – original draft. HWL: Data curation, Investigation, Resources, Writing – original draft, Writing – review & editing. DW: Conceptualization, Formal analysis, Resources, Supervision, Writing – original draft, Writing – review & editing.
